# The Expression Profiles of ADME Genes in Human Cancers and Their Associations with Clinical Outcomes

**DOI:** 10.3390/cancers12113369

**Published:** 2020-11-13

**Authors:** Dong Gui Hu, Peter I. Mackenzie, Pramod C. Nair, Ross A. McKinnon, Robyn Meech

**Affiliations:** Department of Clinical Pharmacology and Flinders Cancer Centre, Flinders University, College of Medicine and Public Health, Flinders Medical Centre, Bedford Park, Adelaide 5042, SA, Australia; peter.mackenzie@flinders.edu.au (P.I.M.); pramod.nair@flinders.edu.au (P.C.N.); ross.mckinnon@flinders.edu.au (R.A.M.); robyn.meech@flinders.edu.au (R.M.)

**Keywords:** cancer, drug absorption, drug distribution, drug metabolism, drug excretion, endobiotic metabolism, overall survival, therapeutic target, prognostic biomarker

## Abstract

**Simple Summary:**

There are roughly 300 genes that are classically defined as ADME genes based on their roles in drug absorption, distribution, metabolism, and excretion (ADME). The expression profiles of ADME genes in human cancers and their impact on cancer patient survival remain to be systematically assessed. Our pan-cancer gene expression analysis revealed that about half of all ADME genes were expressed in all 21 cancers assessed. Most genes showed highly variable expression within and among different cancers. Our pan-cancer survival analysis identified a set of core ADME genes whose intratumoral expression was associated with overall survival in these cancers. These findings highlight the potential implication of ADME genes as cancer prognostic biomarkers and therapeutic targets. We propose that intratumoral expression of ADME genes can influence cancer patient survival through not only drug metabolism and disposition, but also metabolism and disposition of numerous endogenous molecules that can fuel and/or stimulate cancer growth.

**Abstract:**

ADME genes are a group of genes that are involved in drug absorption, distribution, metabolism, and excretion (ADME). The expression profiles of ADME genes within tumours is proposed to impact on cancer patient survival; however, this has not been systematically examined. In this study, our comprehensive analyses of pan-cancer datasets from the Cancer Genome Atlas (TCGA) revealed differential intratumoral expression profiles for ADME genes in 21 different cancer types. Most genes also showed high interindividual variability within cancer-specific patient cohorts. Using Kaplan-Meier plots and logrank tests, we showed that intratumoral expression levels of twenty of the thirty-two core ADME genes were associated with overall survival (OS) in these cancers. Of these genes, five showed significant association with unfavourable OS in three cancers, including SKCM (*ABCC2*, *GSTP1*), KIRC (*CYP2D6*, *CYP2E1*), PAAD (*UGT2B7*); sixteen showed significant associations with favourable OS in twelve cancers, including BLCA (*UGT2B15*), BRCA (*CYP2D6*), COAD (*NAT1*), HNSC (*ABCB1*), KIRC (*ABCG2*, *CYP3A4*, *SLC22A2*, *SLC22A6*), KIRP (*SLC22A2*), LIHC (*CYP2C19*, *CYP2C8*, *CYP2C9*, *CYP3A5*, *SLC22A1*), LUAD (*SLC15A2*), LUSC (*UGT1A1*), PAAD (*ABCB1*), SARC (*ABCB1*), and SKCM (*ABCB1, DYPD*). Overall, these data provide compelling evidence supporting ADME genes as prognostic biomarkers and potential therapeutic targets. We propose that intratumoral expression of ADME genes may impact cancer patient survival by multiple mechanisms that can include metabolizing/transporting anticancer drugs, activating anticancer drugs, and metabolizing/transporting a variety of endogenous molecules involved in metabolically fuelling cancer cells and/or controlling pro-growth signalling pathways.

## 1. Introduction

Cancer is a major public health burden worldwide and is the second leading cause of death after heart disease in many countries [1,2]. The global rise in cancer incidence is not fully understood but is presumably related to population aging and a growing prevalence of cancer risk factors such as alcohol consumption, exposure to industrial pollutants, tobacco use, obesity, and physical inactivity [2]. Using the global data for cancer incidence and mortality produced by the International Agency for Research on Cancer, the Global Burden of Cancer (GLOBOCAN) study predicted 18.1 million cancer cases and 9.6 million cancer deaths worldwide in 2018 [3]. With the advent of genome-wide high-throughput platforms (e.g., microarrays, DNA/RNA deep-sequencing), numerous molecular biomarkers (e.g., causative, prognostic, predictive, and diagnostic) have been discovered for a wide variety of cancers over the last two decades [4,5,6,7]. Such biomarkers not only improve our understanding of cancer development and progression but also facilitate early diagnosis of cancer and accurate prediction of treatment response and clinical outcomes.

ADME genes are a group of genes that are involved in drug absorption, distribution, metabolism, and excretion (ADME) [8,9,10,11]. Currently, the PharmaADME consortium classifies 298 ADME genes that encode phase I (functionalization) and phase II (conjugation) drug-metabolizing enzymes, transporters, and modifiers (http://www.pharmaadme.org) [8,9]. Typical Phase I enzymes are oxidases, hydrolases, dehydrogenases, and deaminases. Cytochrome P450 enzymes (CYPs) such as CYP2A6, CYP2B6, CYP2C8, CYP2C9, CYP2C19, CYP2D6, CYP2E1, CYP3A4, and CYP3A5 are the most important Phase I drug-metabolizing enzymes [12]. Phase II drug-metabolizing enzymes are mainly transferases, such as UDP-glucuronosyltransferases (UGTs), glutathione transferases (GSTs), sulfotransferases (SULTs), N-acetyl transferases (NATs), and thiopurine methyltransferase (TPMT) [13]. Phase I enzymes introduce reactive or polar groups (e.g., hydroxyl) into substrates that are frequently conjugated by phase II enzymes. The resulting products are generally inactive and water-soluble, thus facilitating their excretion from the body through the bile, urine, or feces. Therefore, the combined action of Phase I and II enzymes usually facilitates drug metabolism and clearance [12,14,15]. Drug transporters include the ATP-binding cassette (ABC) transporters and the solute carrier (SLC) transporters. The ABC transporters are mainly efflux transporters (e.g., ABCB1, ABCC2, ABCG2) that extrude drugs out of cells whereas the SLC transporters are influx transporters (e.g., SLC15A2, SLC22A1, SLC22A2, SLC22A6, SLCO1B1, and SLCO1B3) that are involved in the uptake of drugs into cells [16]. In addition to drugs, ADME genes have important roles in metabolizing, transporting, and detoxifying a wide range of endobiotics (e.g., steroid hormones, amino acids, fatty acids, bile acids, lactate) and xenobiotics (e.g., dietary constituents, pollutants, and carcinogens) [14,15,17,18]. Many of these endobiotics and xenobiotics can influence the initiation and progression of cancer via direct effects such as DNA damage, and/or controlling growth signalling pathways. Consistent with the critical roles of ADME genes in metabolizing and clearing anticancer drugs and cancer-modulating compounds, numerous genetic polymorphisms (e.g., single nucleotide polymorphism, SNP) of ADME genes are known to be associated with carcinogenesis and drug response [19,20,21]. However, these genes also show tissue-specific and dynamically-regulated interindividual variation in expression that may have more profound impacts on ADME properties of tissues (including tumours) than genetic variance. ADME genes are highly expressed in the liver, the major organ for systemic drug metabolism and clearance, but are also expressed extrahepatically and in cancerous tissues where their expression has been associated with cancer progression and anticancer drug resistance [19,22,23,24]. This knowledge emphasizes the need for a comprehensive analysis of ADME gene expression profiles in tumours, and their impact on cancer patient survival.

The internationally coordinated 10-year (2006–2015) Cancer Genome Atlas (TCGA) program provides freely-accessible databases for genome-wide molecular profiles (e.g., genomic, transcriptomic, and epigenetic) for over 20,000 primary cancer patients representing 33 different cancer types (https://gdc.cancer.gov). Analyses of these data have greatly advanced our understanding of cancer biology [25,26,27,28,29,30,31,32]. The TCGA project also collected clinicopathological data from cancer patients that allows association analyses between clinical outcomes and molecular profiles, thus facilitating the identification of prognostic and diagnostic biomarkers. The TCGA Pan-Cancer Clinical Data Resources (TCGA-CDR) recently comprehensively assessed the TCGA clinical data and provided strong support for their value for reliable survival analyses using different clinical outcome endpoints: overall survival (OS), disease-specific survival, disease-free interval, and the progression-free interval [33]. Indeed, using TCGA survival data and matched gene expression (RNA-seq) data in tumours, a recent analysis identified all candidate prognostic genes associated with OS for 17 TCGA cancer types [34]. A similar study revealed genes associated with OS for 21 TCGA cancer types [35].

We recently reported the expression profiles of ADME genes and patterns of dysregulation in the TCGA hepatocellular carcinoma cohort (LIHC) [8]. ADME gene expression profiles and their potential dysregulation in cancers other than LIHC are largely unknown; furthermore, no studies have comprehensively assessed the impact of intratumoral ADME gene expression on patient survival. In the present study, we examined the expression profiles of ADME genes in 21 TCGA cancer types and assessed their potential association with patient survival.

## 2. Results

### 2.1. The Expression Profiles of ADME Genes in Human Cancers

Table 1 lists the 21 TCGA cancer types and the number of patients for each of these cancers that were analysed in this study. We examined the expression profiles of all 298 ADME genes in these 21 cancers (Table S1). A unique set of ADME genes was expressed in different cancers; LAML (Acute Myeloid Leukemia) expressed the fewest ADME genes (181 genes) while LIHC (Liver Hepatocellular Carcinoma) expressed the most (248 genes) (Table 1). Of the 298 ADME genes, 157 genes (52%) were expressed in all 21 cancers and only 12 genes (4%) (*CYP11B1*, *CYP11B2*, *DHRS7C*, *GPX5*, *GPX6*, *GSTA3*, *GSTA5*, *LOC731356*, *PLGLB1*, *SLCO6A1*, *SULT1C1*, *UGT2B17*) were not expressed in any of the 21 cancer types analysed (Table S1). When considering the 32 core ADME genes specifically, four cancer types (CESC, LAML, SKCM, UCEC) expressed the fewest of these genes (17 genes) while LIHC expressed the most (29 genes) (Table 1).

### 2.2. Variable Expression of ADME Genes within and among Different Types of Cancers

Most ADME genes had highly variable expression in different cancers (Table S1). Interestingly, thirteen core ADME genes (*ABCB1*, *ABCC2*, *ABCG2*, *CYP2D6*, *CYP2E1*, *CYP3A5*, *DPYD*, *GSTP1*, *GSTT1*, *NAT1*, *SLC15A2*, *SULT1A1*, *TPMT*) were expressed in all 21 cancer types. Figure S1 shows their variable expressions in these cancers. Furthermore, most ADME genes showed highly variable expression among patients within individual cancers. This is exemplified by the interindividual variability in the expression of *ABCB1* (multidrug resistance protein 1, MDR1) in 21 cancer types (Figure S2). For example, there was a huge difference in intratumoral *ABCB1* expression level (RSEM) within the TCGA Kidney Renal Papillary Cell Carcinoma (KIRP) ranging from 6.85 to 21,254.47; of the 285 patients, 55 patients had an expression of <1000 whereas 69 patients had an expression of >5000. We hypothesized that such interindividual variability for *ABCB1* and other ADME genes can alter intratumoral drug metabolism and clearance, and hence influence therapeutic efficacy and patient survival. We assessed this possibility using Kaplan-Meier plots and Logrank Tests as described in Section 2.3 and Section 2.4.

### 2.3. Core ADME Genes Were Significantly Associated with Overall Survival Rates in Cancers

We focused on core ADME genes to investigate the impact of ADME genes on overall survival (OS) rates in 20 TCGA cancer types using Kaplan-Meier survival analysis and the logrank test. To avoid false-positive predictions, we excluded LGG (one of the cancers listed in Table 1) from OS analysis as a recent study reported a very large number of genes were correlated to OS in this cancer [35]. Using the significant Bonferroni-corrected cut-off logrank *p*-value of < 0.05, we found that the intratumoral expression levels of 20 of the 32 core ADME genes were significantly associated with OS rates in at least one cancer type (Table 2). Of these genes, five showed significant association with unfavourable OS in three cancers, including SKCM (*ABCC2*, *GSTP1*), KIRC (*CYP2D6*, *CYP2E1*), PAAD (*UGT2B7*); sixteen genes showed significant associations with favourable OS in twelve cancers, including BLCA (*UGT2B15*), BRCA (*CYP2D6*), COAD (*NAT1*), HNSC (*ABCB1*), KIRC (*ABCG2*, *CYP3A4*, *SLC22A2*, *SLC22A6*), KIRP (*SLC22A2*), LIHC (*CYP2C19*, *CYP2C8*, *CYP2C9*, *CYP3A5*, *SLC22A1*), LUAD (*SLC15A2*), LUSC (*UGT1A1*), PAAD (*ABCB1*), SARC (*ABCB1*), and SKCM (*ABCB1, DPYD*). We describe these results in detail below.

#### 2.3.1. Association of Core ADME Genes Coding for Phase I Drug Metabolism Enzymes with OS Rates in Cancers

**Figure 1:**
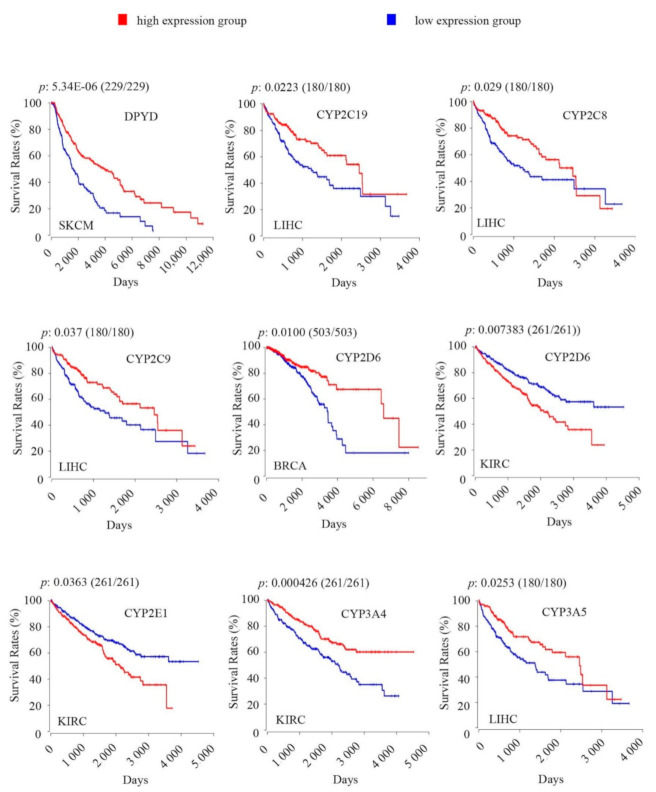
Kaplan-Meier survival analysis and logrank test show significant associations of intratumoral expression levels of core ADME (absorption, distribution, metabolism, and excretion) genes coding for phase I drug-metabolizing enzymes with overall survival rates in TCGA cancers. For Kaplan-Meier survival analysis, the patients were separated into high-expression group (upper 50 percentile, red curve) and low-expression group (lower 50 percentile, blue curve) by gene expression levels in each TCGA cancer type as indicated. The number of patients in each group was given in bracket following the *p*-value. A Bonferroni-corrected cutoff logrank *p*-value of < 0.05 indicates statistical significance.

**Figure 2:**
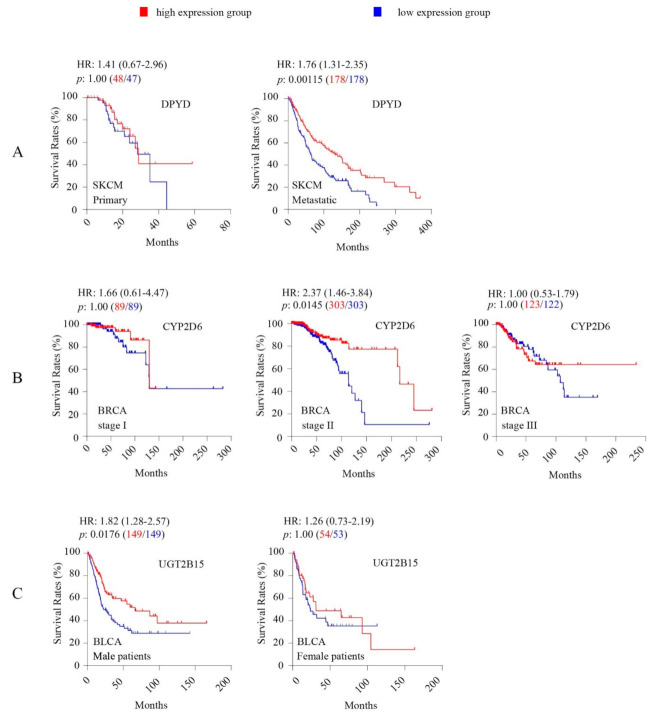
Kaplan-Meier survival analysis and Logrank test show significant associations of intratumoral expression levels of dihydropyrimidine dehydrogenase (DPYD) in skin cancer SKCM (stratified by tumor type) (**A**), CYP2D6 in breast cancer BRCA (stratified by tumor stage) (**B**), and UGT2B15 in bladder cancer (stratified by sex) (**C**) with overall survival rates. For Kaplan-Meier survival analysis, the patients were separated into high-expression group (upper 50 percentile, red curve) and low-expression group (lower 50 percentile, blue curve) by gene expression levels in each TCGA cancer type as indicated. The number of patients in each group was given in bracket following the *p*-value. A Bonferroni-corrected cutoff logrank *p*-value of < 0.05 indicates statistical significance.

Eight core ADME genes coding for phase I drug metabolism enzymes showed significant associations of their intratumoral expression levels with OS rates in cancers, including dihydropyrimidine dehydrogenase *(DPYD)* and seven *CYP* genes (2C19, 2C8, 2C9, 2D6, 2E1, 3A4, 3A5) (Figure 1, Table 2). High intratumoural *DPYD* expression was associated with increased OS rates in SKCM (Figure 1). A recent study reported frequent somatic *DPYD* mutations in SKCM and its upregulation in metastatic tumour [36]. We show here that the association of *DPYD* with favourable OS was seen in metastatic tumours but not in primary tumours (Figure 2A).

Among the CYP genes, four (2C19, 2C8, 2C9, 3A5) showed significant associations with increased OS rates in liver cancer (LIHC); three showed correlation with reduced (2D6, E1) or increased (3A4) OS rates in kidney cancer (KIRC); CYP2D6 was also associated with increased OS rates in breast cancer (BRCA) (Figure 1).

Breast cancers can be classified by a 50-gene signature into five molecular intrinsic PAM50 subtypes: Luminal A, Luminal B, HER2-enriched, Basal-like, and Normal-like [37]. The TCGA BRCA clinical dataset includes records of the PAM50 subtype, tumor stage, and tamoxifen treatment. We stratified the patients by these factors and performed further analysis for *CYP2D6*. The association of *CYP2D6* with favorable OS was seen only in patients with stage II tumour (Figure 2B) but not in any of the PAM50 subtypes (Figure S3B), or within the cohort (248 patients) treated with tamoxifen (Table S3). To assess whether CYP2D6 was differentially expressed between PAM50 subtypes, we performed a one-way ANOVA analysis followed by Tukey’s multiple comparison test. Our results showed higher CYP2D6 expression in the basal-like subtype relative to all other PAM50 subtypes (Figure S3A).

#### 2.3.2. Association of Core ADME Genes Coding for Phase II Drug Metabolism Enzymes with OS Rates in Cancers

**Figure 3:**
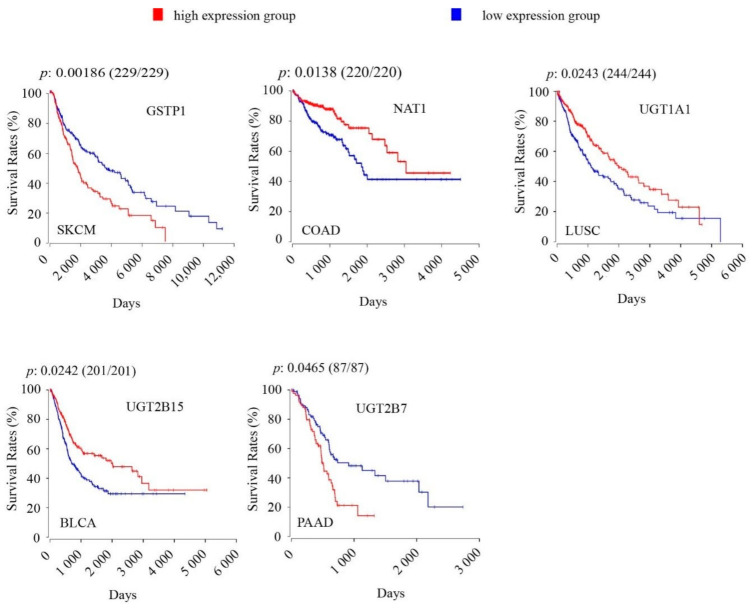
Kaplan-Meier survival analysis and Logrank test show significant associations of intratumoral expression levels of core ADME genes coding for phase II drug-metabolizing enzymes with overall survival rates in TCGA cancers. For Kaplan-Meier survival analysis, the patients were separated into high-expression group (upper 50 percentile, red curve) and low-expression group (lower 50 percentile, blue curve) by gene expression levels in each TCGA cancer type as indicated. The number of patients in each group was given in bracket following the *p*-value. A Bonferroni-corrected cutoff logrank *p*-value of < 0.05 indicates statistical significance.

Five core ADME genes coding for phase II drug metabolism enzymes showed significant associations of their intratumoral expression levels with OS rates in cancers, including *GSTP1*, *NAT1*, *UGT1A1*, *UGT2B15*, *UGT2B7* (Figure 3, Table 2). Of these genes, three showed association with increased OS rates in COAD (*NAT1*), LUSC (*UGT1A1*), or BLCA (*UGT2B15*), whereas the other two genes showed correlation with reduced OS rates in SKCM (*GSTP1*) or PAAD (*UGT2B7*).

Androgen signaling is involved in bladder carcinogenesis and promotes bladder cancer growth [38]. UGT2B15 inactivates androgens and thus represses androgen signaling [39]; hence, we examined whether the association of *UGT2B15* with OS was different in male and female patients. We found that higher expression of *UGT2B15* was associated with increased OS only in male patients (Figure 2C).

#### 2.3.3. Association of Core ADME Genes Coding for Drug Transporters with OS Rates in Cancers

**Figure 4:**
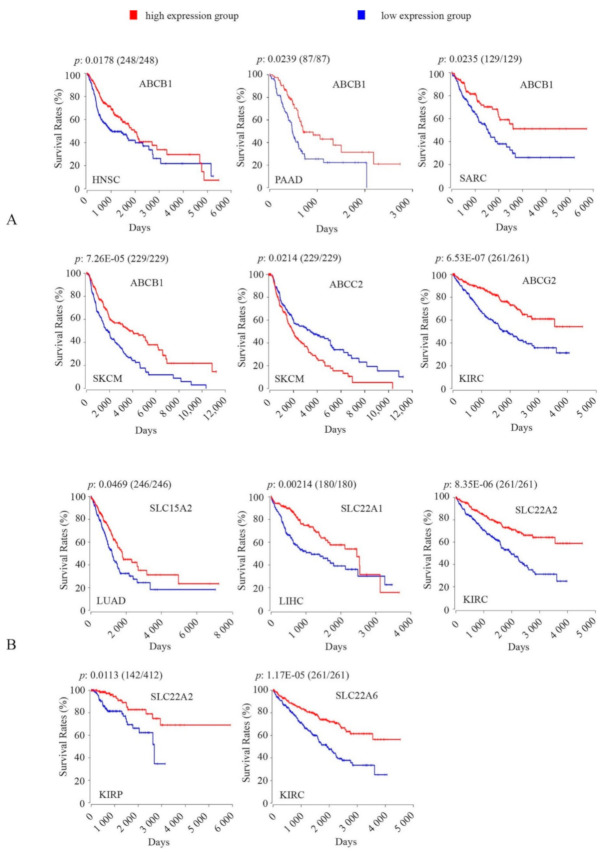
Kaplan-Meier survival analysis and Logrank test show significant associations of intratumoral expression levels of core ADME genes coding for ABC (**A**) and SLC (**B**) drug transporters with overall survival rates in TCGA cancers. For Kaplan-Meier survival analysis, the patients were separated into high-expression group (upper 50 percentile, red curve) and low-expression group (lower 50 percentile, blue curve) by gene expression levels in each TCGA cancer type as indicated. The number of patients in each group was given in bracket following the *p*-value. A Bonferroni-corrected cutoff logrank *p*-value of < 0.05 indicates statistical significance.

Seven core ADME genes coding for drug transporters showed significant associations of their intratumoral expression with OS rates in cancers, including three ABC transporters (B1, C2, G2), and four SLC transporters (15A2, 22A1, 22A2, 22A6) (Figure 4; Table 2). Among the three ABC transporters, high *ABCB1* expression was associated with increased OS rates consistently across four different cancer types (HNSC, PAAD, SARC, SKCM) (Figure 4A); high *ABCG2* expression was also correlated with increased OS rates in KIRC (Figure 4A), consistent with a recent report [40]. By contrast, high *ABCC2* levels showed associations with decreased OS rates in SKCM (Figure 4A).

All of the four SLC transporters showed associations only with increased OS rates in cancers (Figure 4B), including 1) *SLC15A2* (LUAD), *SLC22A1* (LIHC), *SLC22A2* (KIRC, KIRP), and *SLC22A6* (KIRC) (Figure 4B).

### 2.4. Survival Analyses of Core ADME Genes in Non-TCGA Lung and Breast Cancer Datasets

It was not possible to find independent datasets for all of the 20 TCGA cancer types to validate our findings. We were able to assess three non-TCGA cancer datasets from the Kaplan-Meier plotter (KM-LUAD, KM-LUSC, KM-BRCA) [41,42]. As the KM cancer datasets were based on Affymetrix oligo gene expression arrays, many core ADME genes had expression data from more than one probe set. Survival analysis was carried out for all probe sets of every core ADME gene as described in Materials and Methods (Table S3).

**Figure 5:**
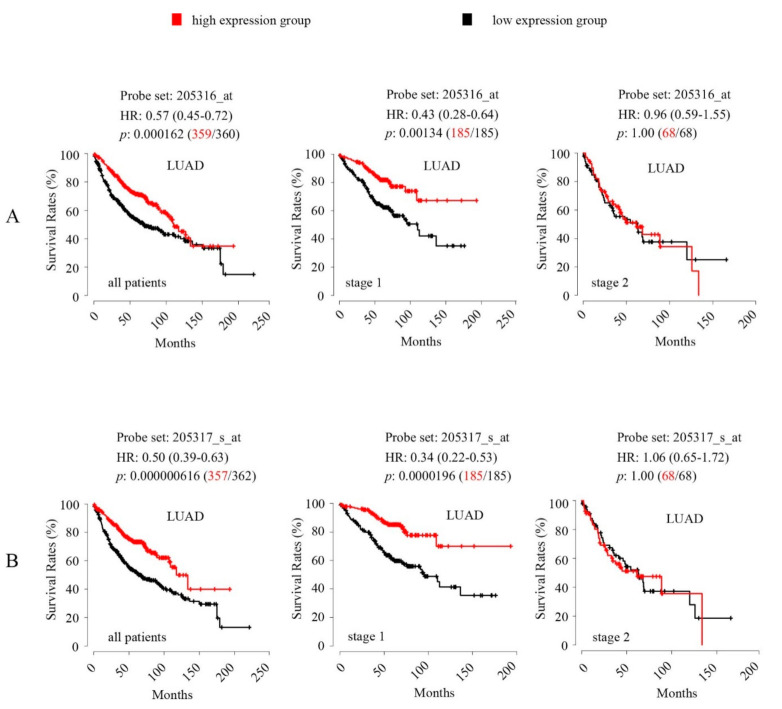
Kaplan-Meier survival analysis and Logrank test show significant associations of intratumoral *SLC15A2* expression levels with overall survival rates in the lung cancer cohort from the Kaplan-Meier Plotter (KM-LUAD). Survival analysis was conducted using the expression data from the two *SLC15A2* probe sets: 205316_at (**A**) and 205317_s_at (**B**). Patients were analysed altogether or following stratification by tumor histology. For analysis, the patients were separated using Median expression into high-expression group (Red curve) and low-expression group (Black curve). The number of patients in each group was given in bracket following the *p*-value. A Bonferroni-corrected cutoff logrank *p*-value of < 0.05 indicates statistical significance. Hazard ratio (HR) and 95% confidence interval (CI) (bracket) are also provided.

All of the core ADME genes that were analysed for the TCGA-LUAD and TCGA-LUSC datasets (Table S3), were also analysed in the KM-LUAD and KM-LUSC datasets. The genes that showed no association with OS in the TCGA datasets also showed no association in the respective KM datasets. The finding that high *SLC15A2* expression associated with favourable OS in the TCGA-LUAD dataset (Figure 4B), was corroborated by the KM-LUAD analysis. Specifically, analysis of the KM-LUAD cohort using expression data from both of the *SLC15A2* probe sets: 205316_at (Figure 5A) and 205317_s_at (Figure 5B), showed a similar association with OS. Stratification by tumor stage indicated that the association was specific to patients with stage I tumour (Figure 5A,B).

High *UGT1A1* expression was associated with favourable OS rates in TCGA LUSC (Figure 3). However, no association was observed in KM-LUSC when expression data from all of the six *UGT1A1* probe sets was examined (Table S3). The *UGT1A* gene superfamily has nine functional isoforms (1A1, 1A3–10), which have a unique exon 1 but share exons 2–5 [43]. Of the six *UGT1A1* probe sets, four (215125_s_at, 207126_x_at, 206094_x_at, 204532_x_at) target exon 5, one (208596_s_at) targets exons 3–5, and the sixth (221304_at) targets *UGT1A8* exon 1 [41]. Hence, none of the *UGT1A1*-designated probe sets on the Affymetrix oligo arrays were *UGT1A1*-specific; this might explain the association of *UGT1A1* with OS in the RNAseq-based TCGA-LUSC dataset was not reproducible in the KM-LUSC dataset.

All of the core ADME genes that were analysed in the TCGA-BRCA dataset (Table S3), were also analysed in the KM-BRCA dataset (Table S3). All but one of the ADME genes that showed no association with OS in the TCGA-BRCA data also showed no association in the KM-BRCA dataset (Table S3). The exception was *GSTM1*, where higher expression was associated with favourable OS in KM-BRCA when analysed using data from both *GSTM1* probe sets (215333_x_at, 204550_x_at) (Figure S4). Moreover, our finding that high *CYP2D6* levels associated with favourable OS in the TCGA-BRCA dataset (Figure 1), was not corroborated by the KM-BRCA analysis when all three *CYP2D6* probe sets were analysed (207498_s_at, 215809_at, 217468_at) (Table S3) [41].

Overall, these data provide confirmation for most of the genes analysed from the three TCGA cancer datasets, but also provide conflicting results for two genes (*CYP2D6*, *GSTM1*) from the KM-BRCA dataset. Our results also point out the limitation of the Affymetrix oligo arrays-based gene expression data to study genes with probe sets that cross-hybridize to other genes. Clearly, future RNAseq-based studies (similar to TCGA projects) will require to verify the findings of this study and many other studies that have analysed the TCGA cancer datasets [34].

## 3. Discussion

Using OncoLnc [35], we comprehensively assessed for the first time the expression profiles of ADME genes in 21 TCGA cancer types. Approximately half of all ADME genes were expressed in all cancer types, with very variable expression within and among different cancers. The widespread and high intratumoral expression of ADME genes is consistent with the view that factors controlling the levels/activities of drugs and other small molecules are not restricted to detoxification organs such as liver, and emphasizes the capacity of tumours to control ADME activities. We also comprehensively assessed potential associations between intratumoral expression of core ADME genes and overall survival (OS) in 20 TCGA cancer types. Overall, twenty core ADME genes showed significant associations with OS in at least one cancer (Table 2). Most of these genes were associated with favourable OS. Eleven of the 25 significant associations reported by the present study were consistent with the results from previous reports, including the Human Protein Atlas [34], the Kaplan-Meier plotter [44], and many others cited below. Further analyses of the KM-LUAD dataset validated our findings from the TCGA-LUAD dataset. Taken together, our results demonstrate abundant expression of ADME genes in human cancers and their potential implications as prognostic biomarkers or therapeutic targets.

Many phase I and II drug-metabolizing enzymes (e.g., CYP enzymes, aldehyde oxidase, glutathione S-transferase) can activate anticancer drugs/prodrugs [45]. Particularly, CYP enzymes are involved in the activation of many commonly used anticancer drugs/prodrugs, including tamoxifen, cyclophosphamide, dacarbazine, trofosfamide, ifosfamide, AQ4N, ftorafur (tegafur), mitomycin C, and flutamide [45,46,47]. Elevated expression of *CYP* genes within cancer cells may increase intracellular concentrations of active drug metabolites, enhancing therapeutic efficacy, and improving cancer patient survival. For example, tamoxifen, a selective estrogen receptor (ER) modulator for treating ER-positive breast cancer, is converted by CYP2D6 to the more potent metabolite 4-hydroxytamoxifen. Our observed association of high intratumoral CYP2D6 expression with favourable OS in breast cancer (BRCA) might be related to its activation of tamoxifen. This finding is in accordance with pharmacogenomic data indicating that patients with functional *CYP2D6* alleles had more favourable clinical outcomes than patients with reduced-function *CYP2D6* alleles following tamoxifen therapy [48]. In LIHC, we showed an association of four *CYP* genes (2C19, 2C8, 2C9, 3A5) with favourable OS (Figure 1). Similar associations with favourable OS in liver cancer have been recently reported for all of these CYP genes [49,50,51,52,53]. Unfortunately, drug regimens were available for only 42 of the 360 LIHC patients (Table S2), and the main drug received was Sorafenib (29 patients), which is not known to be a substrate of these specific CYP enzymes. Moreover, the drug-treated group was too small for robust statistical analysis. In KIRC, *CYP3A4* was associated with favourable OS; by contrast, *CYP2D6* and *CYP2E1* were correlated with unfavourable OS (Figure 1). Drug regimens were available for 83 of the 523 KIRC patients (Table S2). The main drugs received were kinase inhibitors, such as sorafenib, sunitinib, gefitinib, pazopanib, and temsirolimus. Most of these drugs are CYP3A4 substrates [12,54], but none are known CYP450-activated prodrugs. Moreover, because the drug regimens were only available for a small proportion of the patients in KIRC, we were not able to assess whether the association of these *CYP* genes with OS could be related to specific drug regimens.

Phase I and II drug-metabolizing enzymes are also involved in the metabolism and biotransformation of a wide range of endogenous bioactive molecules (e.g., steroid hormones, amino acids, fatty acids, bile acids, lactate) that can fuel or stimulate cancer growth. For example, the two natural androgens, testosterone, and dihydrotestosterone, are primarily inactivated through glucuronidation by three UGT enzymes (2B7, 2B15, 2B17) [39]. There is emerging evidence that androgen signalling is involved in bladder carcinogenesis and progression [38,55,56]. Targeting androgen signalling represents a potential novel therapy for bladder cancer [38]. In BLCA, we showed an association of high *UGT2B15* expression with favourable OS, consistent with a recent report [34]. None of the main drugs (carboplatin, cisplatin, gemcitabine) received by BLCA patients (Table S2) were UGT2B15 substrates. Hence, we hypothesized that UGT2B15 might instead influence BLCA survival by modulating the androgen signalling pathway through androgen conjugation. Bladder cancer occurs more frequently in men than women [38]. Consistently, stratifying the patients by sex revealed that the association of *UGT2B15* with favourable OS occurred only in male patients (Figure 2C). Similar mechanisms whereby UGT2B15 and UGT2B17 influence survival through modulating the androgen signalling pathway have been reported in androgen-sensitive prostate and breast cancer [57,58].

Phase I and II drug metabolizing enzymes may also impact cancer patient survival, independently of their enzymatic activities. For example, CYP3A5 has been reported as a tumour suppressor that inhibits liver cancer cell migration and invasion through suppressing ROS/mTORC2/p-AKT kinase signaling [59]. Low *CYP3A5* expression was correlated with aggressive vascular invasion, poor differentiation, and poor survival in liver cancer [59]. In LIHC, we showed the association of high *CYP3A5* expression with favourable OS (Figure 1), which is consistent with the reported tumour suppressive activity of this enzyme. By contrast, GSTP1 has been reported to possess pro-oncogenic activity that protects cancer cells from apoptosis signals by suppressing MAPK/JNK kinase signaling [60]. In SKCM, we showed a correlation of *GSTP1* with unfavourable OS (Figure 3); however, none of the main drugs (dacarbazine, interferon, ipilimumab) received by SKCM patients were GSTP1 substrates (Table S2). Hence, GSTP1 might modulate OS in SKCM via the reported non-enzymatic pro-oncogenic activity of this enzyme.

The influx transporters are involved in the uptake of anticancer drugs into cells, and therefore their high intratumoral expression may increase therapeutic efficacy and improve cancer patient survival [61,62,63,64]. Indeed, we showed here that four influx transporters (SLC15A2, SLC22A1, SLC22A2, SLC22A6) whose high intratumoral expression was correlated only with favourable OS (Figure 4B). SLC22A2 and SLC22A6 were associated with favourable OS in KIRC (Figure 4B). A similar association of SLC22A6 with favourable OS in kidney cancer (KIRC) was recently reported [65]. SLC22A2 substrates include cisplatin, oxaliplatin, picoplatin, imatinib, irinotecan, paclitaxel, mitoxantrone. SLC22A6 substrates include methotrexate and bleomycin [64]. While KIRC patients received a wide range of drugs (Table S2), none are substrates for SLC22A2 or SLC22A6. *SLC22A2* was also associated with favourable OS in KIRP (Figure 4B); however, drug regimens were only available for 22 of the 285 KIRP patients (Table S2). Finally, we showed an association of *SLC15A2* with favourable OS in TCGA LUAD; this was consistent with a recent report [66], and we also corroborated this finding in the KM-LAUD dataset (Figure 5B). SLC15A2 is a peptide transporter (PEPT2) involved in transport of numerous di-and tripeptides, and peptidomimetic drugs (e.g., delta-aminolevulinic acid (δ-ALA), fosinopril] [67]). However, none of the main drugs (carboplatin, cisplatin, gemcitabine, pemetrexed, taxol) received by the LUAD patients were SLC15A2 substrates (Table S2). The association of this gene with favourable OS in LUAD might be related to the transport of endogenous oligopeptides; this hypothesis requires further investigation.

The efflux transporters (e.g., ABCB1, ABCC2, ABCG2) extrude anticancer drugs out of cells and confer multi-drug resistance [24,68]. Studies have shown that their high expression within the tumour was correlated to poor drug response and unfavourable cancer patient survival [69,70]. We showed here that high *ABCC2* expression was associated with unfavourable OS in melanoma (SKCM) (Figure 4A). Drug regimens were available for only 142 of the 459 SKCM patients, and the range of drugs was very broad, with no individual drugs received by more than 20% of patients (Table S2). Some of these drugs (e.g., cisplatin, melphalan, vinblastine) were ABCC2 substrates but were received by less than 20 patients [71]. Hence, the association of *ABCC2* with unfavourable OS in SKCM could not be related to drug efflux.

The most intriguing finding was the association of efflux transporters ABCB1 and ABCG2 with favourable OS in cancers (Figure 4A). We showed an association of *ABCG2* with favourable OS in KIRC, consistent with a recent report [40]. Many of the drugs received by KIRC patients were ABCG2 substrates, such as sorafenib, sunitinib, gefitinib, pazopanib, and temsirolimus (Table S2) [71]. As drug regimens were only available for 83 of the 523 KIRC patients, we were not able to assess any relationship between this association and drug regimens. We showed a correlation of *ABCB1* with favourable OS in four separate cancer types (HNSC, PAAD, SARC, SKCM) (Figure 4A). Similar associations of *ABCB1* with favourable OS have been reported in two of these cancers (HNSC, PAAD) [34,72,73,74,75], and also in breast cancer (BRCA) [76] and kidney cancer (KIRC) [34,77]. High *ABCB1* expression was also associated with favourable OS in neuroblastoma [78,79]. Our findings together with these reports strongly argue that ABCB1 has a positive impact on cancer patient survival. None of the main drugs received by PAAD or SKCM patients were ABCB1 substrates (Table S2); therefore, the association of *ABCB1* with favourable OS could not be attributed to drug efflux in these two cancers. Considering HNSC and SARC, paclitaxel was received by 36 of the 497 HNSC patients and doxorubicin was received by 38 of the 259 SARC patients; both drugs are ABCB1 substrates [71]. However, this small proportion of the patients treated with these drugs limits any analysis of the relationship between the association and drug regimens. Taken together, these observations led us to hypothesize that ABCB1 may influence cancer patient survival independently of drug efflux. In addition to drug efflux, ABCB1 is also involved in transporting a variety of endogenous substrates, such as phospholipids (sphingomyelin, glucosylceramide), steroid hormones and their metabolites (glucocorticoids, aldosterone, β-estradiol-glucuronide), cytokines (IL-1β, IL-2, IL-4, IFNγ), and platelet-activating factor (PAF) [80,81,82]. ABCB1 also has an important role in regulating programmed cell death, apoptosis [82]. Therefore, ABCB1 may positively influence cancer patient survival by exporting these and other unknown endogenous substrates that may fuel or stimulate cancer growth. Most clinical trials of inhibitors targeting ABCB1 resulted in no benefit in survival [24]. This raises the question of how significant ABC transporters are in contributing to clinical drug resistance [83]. A recent call for the re-evaluation of their role in mediating multidrug-resistance has revitalized this debate [24]. Our findings that *ABCB1* was associated with favourable survival in multiple cancers emphasizes the need for further studies to clarify whether ABCB1 should be targeted for cancer therapy.

The TCGA projects have defined the comprehensive molecular profiles (e.g., genomic, transcriptomic, epigenetic, proteomic) for common human cancers and discovered numerous therapeutic targets and causative, diagnostic, or prognostic biomarkers [34]. However, we recognize the limitations of the TCGA datasets to decipher drug-specific mechanisms. A major limitation is that tumour samples were obtained prior to therapeutic intervention. Many ADME genes (e.g., CYPs and UGTs) are induced by common cytotoxic anticancer drugs [84,85,86]. Inter-individual differences in ADME gene induction might lead to different drug responses; however, testing this hypothesis would require tumour samples to be sampled during/after treatment. Another limitation is that information on drug regimens was typically only available for a small proportion of the patients for most cancer types. Furthermore, drug type, dose, and/or treatment duration varied among patients, and most patients received multiple therapy types, including chemotherapy, hormone therapy, ancillary therapy, vaccine, immunotherapy, or targeted therapy. Such diverse drug regimens challenge our ability to define drug-specific mechanisms. Given these limitations, future purpose-designed prospective studies with sufficient patients and potentially incorporating longitudinal tumour sampling will be required to precisely assess gene-/drug-specific impacts on cancer patient survival.

## 4. Materials and Methods

### 4.1. ADME Genes and TCGA Cancer Types

Roughly 300 genes are considered as ADME genes with slightly different lists among studies [8,9,10,11,87]. The present study assessed the 298 ADME genes defined by the PharmaADME Consortium (http://www.pharmaadme.org) as previously reported [8,9]. These 298 genes include 32 core ADME genes and 266 extended ADME genes (Table S1). Core ADME genes are the most important genes directly involved in drug metabolism and clearance; the extended ADME genes are other genes related to drug metabolism/clearance. ADME genes are categorized into three groups: 1) phase I and II enzymes; 2) transporters; 3) modifiers (modulating the expression or activity of other ADME genes). The full names for all 298 ADME genes are given in Table S1.

Table 1 lists the 21 TCGA cancer types (7983 patients in total) that were included in our analysis of ADME gene expression profiles. For 20 of these cancer types, only primary tumours were included in the analysis; the SKCM dataset included about 30% primary tumour and 70% metastatic tumours [35].

The drug regimens of TCGA cancer types (Table S2) were downloaded from the GDC (Genomic Data Commons) data portal (https://portal.gdc.cancer.gov) using an R/Bioconductor package TCGAbiolinks as previously reported [88,89].

### 4.2. ADME Gene Expression Profiles Among and Within Cancers

The mRNA levels (RNASeqV2) of ADME genes of 21 different TCGA cancer types were obtained as normalized RSEM values from the OncLnc database (http://www.oncolnc.org). RSEM is one of the most frequently used programs for quantifying transcript abundances from RNA-Seq data [90]. Only genes with a median expression higher than 1 RSEM and no more than 25% of the patients with an expression of 0 were included in the analysis [35]. The expression profiles and variable expression of ADME genes within and between cancers were graphed using GraphPad Prism (version 7.03) (GraphPad Software Inc, San Diego, CA, USA).

### 4.3. Assessment of Associations between the Intratumoral Expression Levels of Core ADME Genes and Overall Survival of Cancer Patients Using the OncoLnc Database

The Kaplan-Meier estimator is one of the most frequently used methods for clinical survival analysis [91]. In a clinical setting, patients are often recruited at different stages of the study or even leave the study before its completion. Overall survival (OS) time was defined as the time from the day at diagnosis to the date of death (dead patients) or the date of the last follow-up (censored patients). The Kaplan-Meier estimator allows these “censored data” to be included in the survival analysis. Using this approach, a recent study assessed the genome-wide associations of gene expression and overall survival (OS) for 21 different types of TCGA cancers and established the publicly accessible OncoLnc database for survival analysis of genes of interest for the scientific community using Kaplan-Meier plots and logrank tests [35]. The analysis from this study reported the association of OS with a large number of genes in LGG. To avoid false-positive predictions, we excluded this cancer from our OS analysis. Using the OncoLnc platform, we examined potential associations between intratumoral expression levels of all 32 core ADME genes and OS rates for each of the remaining 20 TCGA cancer types. For the Kaplan-Meier survival analysis, we separated the patients by gene expression into high-expression group (upper 50 percentile) and low-expression group (lower 50 percentile) and performed logrank tests. To control for false discovery rates, we performed multiple testing correction using the R-statistical program (version 3.51) (https://cran.r-project.org). Specifically, we performed Bonferroni correction separately for each of the 20 TCGA cancer types using the raw *p* values of all independent logrank tests conducted from each specific cancer type. As a varying number of core ADME genes were expressed in different cancer types (Table 1), the number of independent logrank tests performed varied among different cancers. The Bonferroni correction is the most stringent test for multiple testing correction and offers the most conservative approach to minimize false-positive discovery rates [92]. A Bonferroni-corrected cutoff logrank *p*-value of < 0.05 was considered to be statistically significant. Table S3 lists both raw and Bonferroni-corrected *p* values for independent logrank tests of all core ADME genes that were conducted for each of the 23 cancer types, including 20 TCGA cancers and 3 non-TCGA cancers (KM-LUAD, KM-LUSC, KM-BRCA). Of note, the Bonferroni correction was conducted separately for each of these cancer types, not for study-wide comparisons.

The OncoLnc survival analysis tool is not able to stratify patients using clinical parameters such as sex, tumor stage, and subtype. For analysis of three genes (*CYP2D6* in BRCA, *DPYD* in SKCM, *UGT2B15* in BLCA) stratified by clinicopathological parameters (as indicated in Figure 2 and Figure S3B), we obtained the clinical datasets for these three TCGA cancers and the expression levels for these three genes from respective cancers from the cBioPortal [93] (https://www.cbioportal.org). We performed Kaplan-Meier survival analysis and logrank tests using the GraphPad Prism software (version 7.03). For the analysis, we separated the patients by gene expression into a high-expression group (upper 50 percentile) and a low-expression group (lower 50 percentile). A Bonferroni-corrected cutoff logrank *p*-value of < 0.05 was considered to be statistically significant. The GraphPad Prism software generated Hazard ratio (HR) and 95% confidence interval (CI) for the analysis. Both HR and 95% CI values for relevant survival analyses are given in Figure 2 and Figure S3B.

### 4.4. Survival Analysis of Core ADME Genes in Non-TCGA Cancer Datasets

It was not possible to find independent datasets with transcriptomic profiling and overall survival data for all of the 20 TCGA cancer types analysed in the present study. However, we were able to analyse the lung cancer dataset and the breast cancer dataset from the Kaplan-Meier Plotter (KM-lung cancer) [41] (https://kmplot.com) to validate our findings from the three TCGA cancer types (BRCA, LUAD, LUSC). The KM-lung cancer dataset was established using gene expression data (Affymetrix HGU133A, HG133A+2, and HGU133+2) and clinicopathological parameters of 2437 patients that were collected from 14 published independent datasets (accessed 1 October 2020) [41]. Of these patients, there were 1925 patients with overall survival data for analysis, including 865 patients with adenocarcinoma (KM-LUAD) and 675 patients with squamous cell carcinoma (KM-LUSC). The breast cancer dataset (KM-BRCA) was also established using gene expression data (Affymetrix HGU133A, HGU133+2) and clinical data of 5139 patients that were collected from 35 independent Gene expression Omnibus (GEO) datasets (accessed 1 October 2020, https://kmplot.com) [42]. Of this dataset, there were 1402 patients with overall survival data for analysis.

We plotted the Kaplan-Meier plots and performed the logrank tests for the same set of core ADME genes (Table S3) that were analysed for the three TCGA cancers (BRCA, LUAD, LUSC) through the Kaplan-Meier plotter (https://kmplot.com). As listed in Table S3, most core ADME genes analysed had more than one probe set on the Affymetrix HGU oligo arrays. We performed survival analysis for all probe sets for every core ADME gene analysed. Because of this, we performed a total of 67, 56, and 54 independent logrank tests for the 19, 18, and 23 core ADME genes that were analysed for KM-BRCA, KM-LUAD, and KM-LUSC, respectively (Table S3). Raw independent logrank *p* values of all probe sets conducted for each of the three datasets (KM-LUAD, KM-LUSC, KM-BRCA) were adjusted separately using Bonferroni correction. A Bonferroni-corrected cutoff logrank *p*-value of < 0.05 was considered to be statistically significant. A significant association was defined where all probe sets of a gene had a Bonferroni-corrected *p*-value of < 0.05. Conflicting results were seen for some genes such as *DPYD* in KM-LUAD, where all three *DPYD* probe sets showed a Bonferroni-corrected *p*-value of < 0.05; however, one probe set (1554534_at) was associated with unfavourable OS but the two other probe sets (1554536_at, 204646_at) showed association with favourable OS (Table S3). Genes with conflicting results among their probe sets were considered to be not statistically significant.

The Kaplan-Meier plotter generated Hazard ratio (HR) and 95% confidence interval (CI) for each analysis. Both HR and 95% CI values for the survival analyses using the *SLC15A2* expression levels from its two probe sets in KM-LUAD were given in Figure 5.

## 5. Conclusions

In conclusion, we have defined the expression profiles of ADME genes in 21 TCGA cancer types. We also identified 20 core ADME genes whose intratumoral expression was significantly associated with overall survival in at least one cancer type. Our results provide compelling evidence supporting ADME genes as potential prognostic biomarkers and therapeutic targets. As data on drug treatment regimens were limited, for most TCGA cancer types we were unable to assess whether the associations of ADME genes with survival could be attributed to anticancer drug regimens. Despite this, we have provided the first comprehensive set of evidence that intratumoral ADME gene expression may impact patient survival through a diverse range of mechanisms, including metabolism/transport of anticancer drugs, activation of pro-drugs, metabolism/transport of endogenous molecules that can fuel or stimulate cancer growth, and possible non-enzymatic mechanisms that have recently emerged in the literature.

## Figures and Tables

**Table 1:** This table lists the number of patients analysed, the number of ADME (absorption, distribution, metabolism, and excretion) genes and core ADME genes expressed for each of 21 TCGA cancer types.

Cancer Types	Description	No. of Patients	No. of ADME Genes	No. of Core ADME Genes
BLCA	Bladder Urothelial Carcinoma	403	220	20
BRCA	Breast Invasive Carcinoma	1006	216	19
CESC	Cervical Squamous Cell Carcinoma and Endocervical Adenocarcinoma	264	214	17
COAD	Colon Adenocarcinoma	440	228	25
ESCA	Esophageal Carcinoma	144	218	21
GBM	Glioblastoma Multiforme	153	206	18
HNSC	Head and Neck Squamous Cell Carcinoma	497	215	18
KIRC	Kidney Renal Clear Cell Carcinoma	523	235	23
KIRP	Kidney Renal Papillary Cell Carcinoma	285	225	22
LAML	Acute Myeloid Leukemia	151	181	17
LGG	Brain Lower Grade Glioma	510	213	20
LIHC	Liver Hepatocellular Carcinoma	360	248	29
LUAD	Lung Adenocarcinoma	492	225	18
LUSC	Lung Squamous Cell Carcinoma	489	228	23
OV	Ovary Serous Cystadenocarcinoma	294	219	20
PAAD	Pancreatic Adenocarcinoma	175	243	26
READ	Rectum Adenocarcinoma	159	229	23
SARC	Sarcoma	259	200	18
SKCM	Skin Cutaneous Melanoma	459	196	17
STAD	Stomach Adenocarcinoma	379	229	24
UCEC	Uterine Corpus Endometrial Carcinoma	541	221	17

**Table 2:** Kaplan-Meier survival analyses/logrank tests showed associations of intratumoral expression levels of core ADME genes with favourable or unfavourable overall survival rates in TCGA cancer types.

Core ADME Genes	TCGA Cancers	Adjusted *p*-Value Bonferroni Correction	Prognostic Biomarker
ABCB1	HNSC	0.01788	Favourable
ABCB1	PAAD	0.02392	Favourable
ABCB1	SARC	0.02358	Favourable
ABCB1	SKCM	7.26 × 10^−05^	Favourable
ABCC2	SKCM	0.021470	Unfavourable
ABCG2	KIRC	6.53 × 10^−07^	Favourable
CYP2C19	LIHC	0.02230	Favourable
CYP2C8	LIHC	0.02987	Favourable
CYP2C9	LIHC	0.03712	Favourable
CYP2D6	BRCA	0.0100	Favourable
CYP2D6	KIRC	0.00738	Unfavourable
CYP2E1	KIRC	0.03634	Unfavourable
CYP3A4	KIRC	0.00042	Favourable
CYP3A5	LIHC	0.02534	Favourable
DPYD	SKCM	5.34 × 10^−06^	Favourable
GSTP1	SKCM	0.00186	Unfavourable
NAT1	COAD	0.01382	Favourable
SLC15A2	LUAD	0.04690	Favourable
SLC22A1	LIHC	0.00214	Favourable
SLC22A2	KIRC	8.35 × 10^−06^	Favourable
SLC22A2	KIRP	0.01139	Favourable
SLC22A6	KIRC	1.17 × 10^−05^	Favourable
UGT1A1	LUSC	0.02438	Favourable
UGT2B15	BLCA	0.02420	Favourable
UGT2B7	PAAD	0.04654	Unfavourable

## Supplementary Materials

The following are available online at https://www.mdpi.com/2072-6694/12/11/3369/s1, Figure S1: The variable expression profiles of thirteen core ADME genes in 21 different types of TCGA cancers; Figure S2: The inter-patient variable expression profiles of *ABCB1* in 21 different types of TCGA cancers; Figure S3: Expression and survival analyses of CYP2D6 in TCGA BRCA subtypes; Figure S4: Significant association between intratumoral *GSTM1* expression levels and overall survival in the KM-BRCA cohort; Table S1: The complete lists of ADME genes and their expression levels in 21 different types of TCGA cancers; Table S2: Drug regimens of TCGA cancer types; Table S3: Multiple testing corrections using Bonferroni correction.
