# Supplementary material for: The Expression Profiles of ADME Genes in Human Cancers and Their Associations with Clinical Outcomes
Source: Cancers (Basel). 2020 Nov 13;12(11):3369. doi: 10.3390/cancers12113369 (PMC7697355; doi:10.3390/cancers12113369)
Supplement: Supplementary file 1 [file cancers-12-03369-s001.zip › cancers-942388 Supplementary Material.pdf]

Supplementary Material: The Expression Profiles of ADME Genes in Human Cancers and Their Associations with Clinical Outcomes

Dong Gui Hu, Peter I. Mackenzie, Pramod C. Nair, Ross A. McKinnon and Robyn Meech

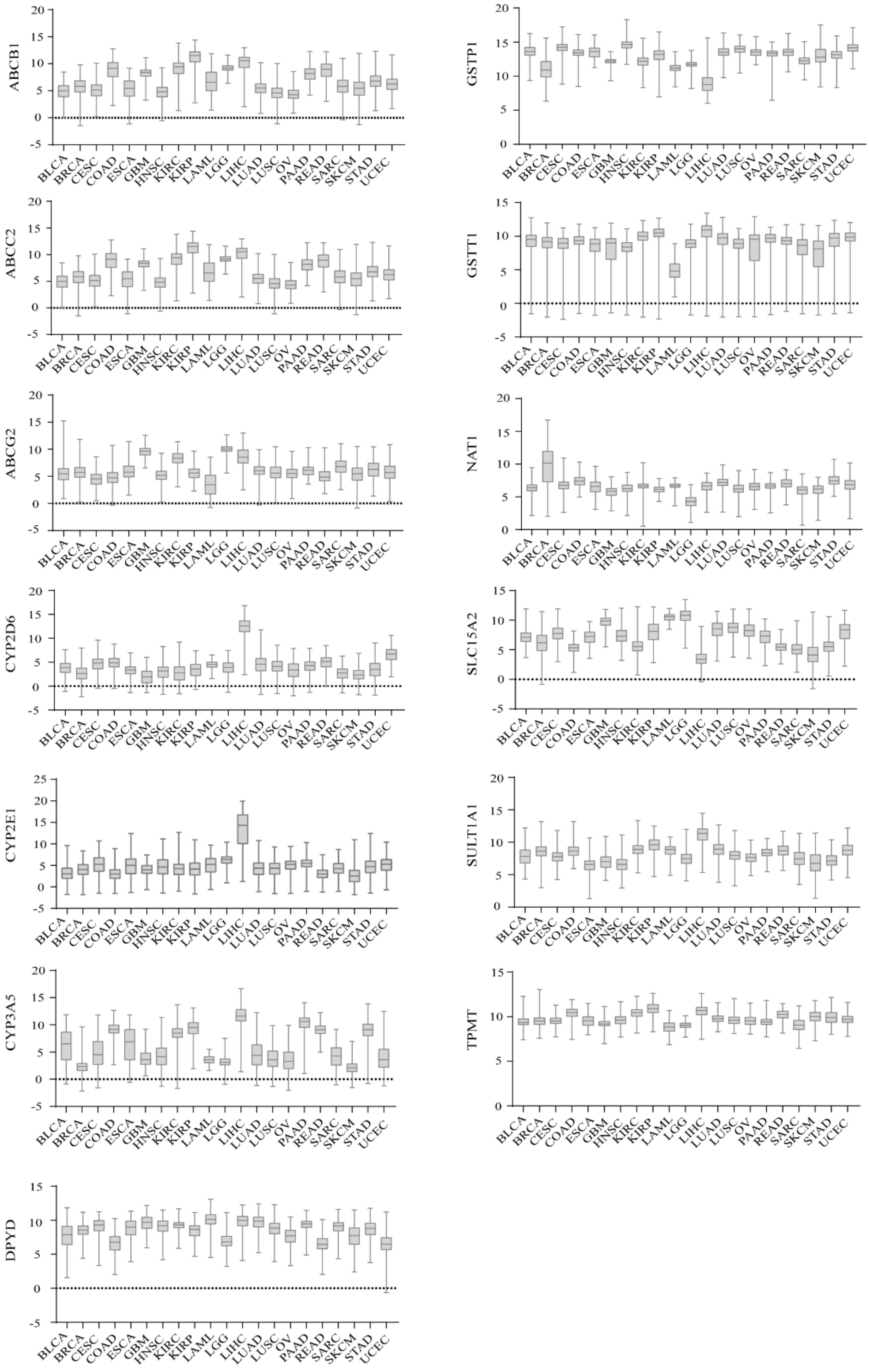

Figure S1. The variable expression profiles of thirteen core ADME genes in 21 different types of TCGA cancers. The log2-transformed expression levels (RSEM values) of thirteen core ADME genes in 21 TCGA cancer types are presented using the box-and-whisker plots that show the distribution of the expression levels (minimum, first quartile, median, third quartile, and maximum) in each of these cancers.

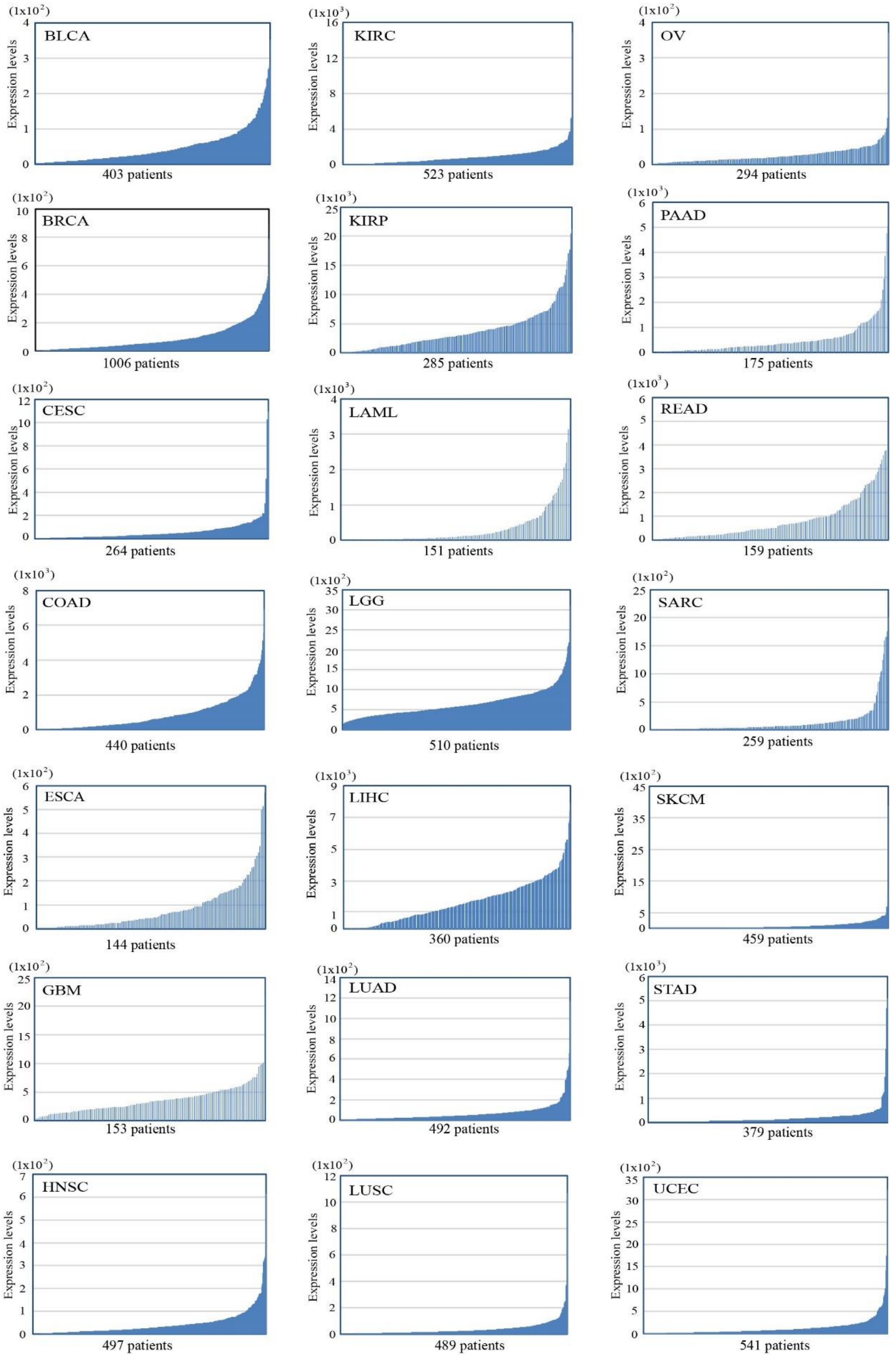

Figure S2. The inter-patient variable expression profiles of *ABCB1* in 21 different types of TCGA cancers. The log2-transformed expression levels (RSEM values) of ABCB1 in 21 cancer types are presented from the minimum to maximum expression level for all patients in each of these cancers.

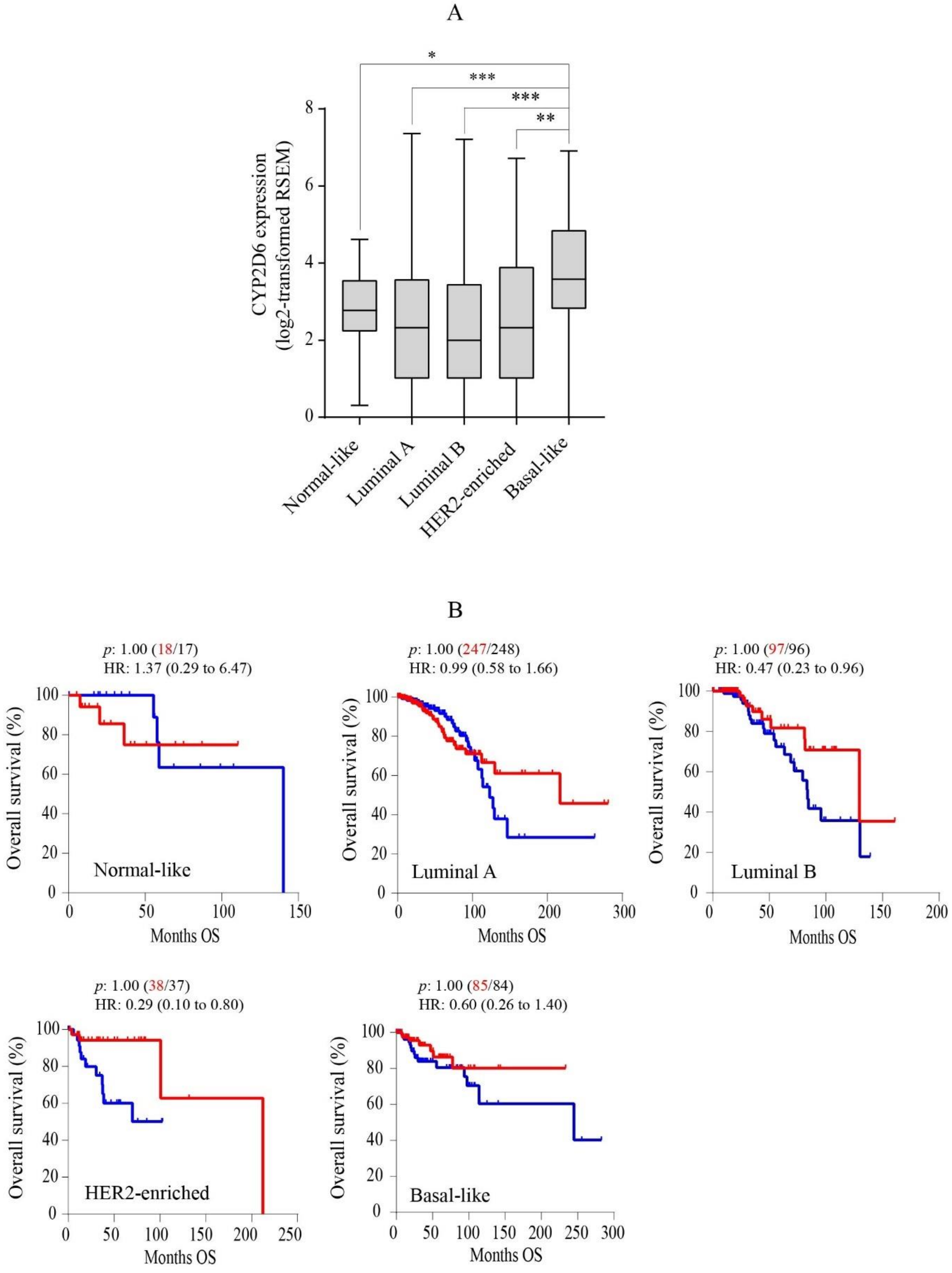

Figure S3. Expression and survival analyses of CYP2D6 in TCGA BRCA subtypes. (**A**) The box-and-whisker plot shows the log2-transformed CYP2D6 expression levels (RSEM values) (minimum, first quartile, median, third quartile, and maximum) in five PAM50 subtypes. One-way ANOVA analysis was followed by Tukey's multiple comparisons test. * $p < 0.05$; ** $p < 0.01$; *** $p < 0.001$. (**B**) Survival

analyses of CYP2D6 expression using Kaplan-Meier survival analysis and logrank test. A Bonferroni-corrected cutoff logrank *p* value of < 0.05 indicates statistical significance. The patients were separated into high-(red curve) and low (blue curve)-expression groups using the median of the CYP2D6 expression level. The number of patients in each group was given in bracket following the *p* value. HR: Hazard ratio; CI: confidence interval.

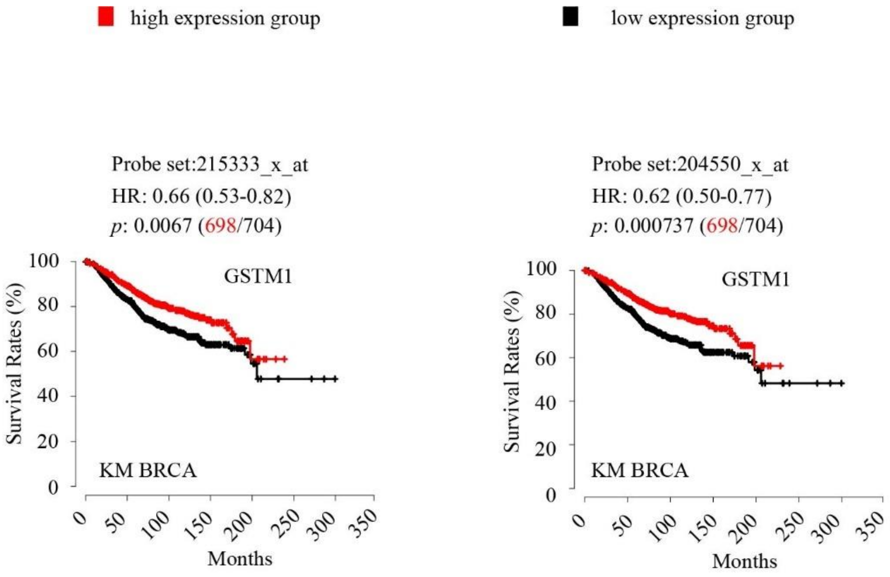

Figure S4. Significant association between intratumoral *GSTM1* expression levels and overall survival in the KM-BRCA cohort. The analysis was conducted for the two *GSTM1* probe sets (215333_x_at, 204550_x_at) on the Affymetrix oligo arrays that showed consistent results. For the analysis, the patients were separated into high-(red curve) and low (black curve)-expression groups using median CYP2D6 expression levels. A Bonferroni-corrected cutoff logrank *p* value of < 0.05 indicates statistical significance. The number of patients in each group was given in bracket following the p value. Hazard ratio (HR) and 95% confidence interval (CI) ware also given.

Table S1 (separate Excel file "Table S1"): The complete lists of ADME genes and their expression levels in 21 different types of TCGA cancers.

Table S2 (separate Excel file "Table S2"): Drug regimens of TCGA cancer types.

Table S3 (separate Excel file "Table S3"): Multiple testing corrections using Bonferroni correction.
